# Validation of an anxiety scale for prenatal diagnostic procedures

**DOI:** 10.11606/S1518-8787.2019053000621

**Published:** 2019-01-18

**Authors:** Lucas Kindermann, Jefferson Traebert, Rodrigo Dias Nunes

**Affiliations:** I Universidade do Sul de Santa Catarina. Faculdade de Medicina. Palhoça, SC, Brasil; II Universidade do Sul de Santa Catarina. Programa de Pós-Graduação em Ciências da Saúde. Palhoça, SC, Brasil

**Keywords:** Ultrasonography Prenatal, psychology, Test Anxiety Scale, Surveys and Questionnaires, utilization, Translations, Validation Studies, Ultrassonografia Pré-Natal, psicologia, Escala de Ansiedade Frente a Teste, Inquéritos e Questionários, utilização, Traduções, Estudos de Validação

## Abstract

**OBJECTIVE:**

To perform a cross-cultural adaptation of the Prenatal Diagnostic Procedures Anxiety Scale questionnaire for application in the Brazilian cultural context.

**METHODS:**

The translation and back translation processes followed internationally accepted criteria. A committee of experts evaluated the semantic, idiomatic, experimental and conceptual equivalence, proposing a pre-final version that was applied in 10.0% of the final sample. Afterwards, the final version was approved for the psychometric analysis. At that stage, 55 pregnant women participated which responded to the proposed Brazilian version before taking an ultrasound examination at a public hospital in Santa Catarina, in the year of 2017. The Edinburgh Postnatal Depression Scale was used as an external reliability parameter. The internal consistency of the instrument was obtained by Cronbach's alpha. Validation was performed by exploratory factorial analysis with extraction of principal components by the Kaiser-Guttman method and Varimax rotation.

**RESULTS:**

The Cronbach's alpha value of the total instrument was 0.886, and only the percentage of variance from item 2 (0.183) was not significant. The Kaiser-Guttman criterion defined three factors responsible for explaining 78.5% of the variance, as well as the Scree plot. Extraction of the main components by the Varimax method presented values from 0.713 to 0.926, with only item 2 being allocated in the third component.

**CONCLUSIONS:**

The Brazilian version is reliable and valid for use in the diagnosis of anxiety related to the performance of ultrasound procedures in prenatal care. Due to the lack of correlation with the rest of the construct, it is suggested that item 2 be removed from the final version.

## RESUMO

**OBJETIVO:**

Proceder à adaptação transcultural do questionário *Prenatal Diagnostic Procedures Anxiety Scale* para aplicação no contexto cultural brasileiro.

**MÉTODOS:**

Os processos de tradução e retrotradução seguiram critérios aceitos internacionalmente. Um comitê de especialistas avaliou as equivalências semântica, idiomática, experimental e conceitual, propondo uma versão pré-final que foi aplicada em 10,0% da amostra final. Em seguida, foi aprovada a versão final para a análise psicométrica. Nessa etapa participaram 55 gestantes que responderam à versão brasileira proposta antes de realizarem um exame ultrassonográfico em um hospital público de Santa Catarina, no ano de 2017. A *Edinburgh Postnatal Depression Scale* foi utilizada como parâmetro de confiabilidade externa. A consistência interna do instrumento foi obtida pelo alfa de Cronbach. A validação foi realizada por análise fatorial exploratória com extração de componentes principais pelo método de Kaiser-Guttman e rotação Varimax.

**RESULTADOS:**

O alfa de Cronbach do instrumento total foi 0,886, e apenas o percentual de variância do item 2 (0,183) não foi significativo. O critério de Kaiser-Guttman definiu três fatores responsáveis por explicar 78,5% da variância, assim como o gráfico de Escarpa. A extração dos componentes principais pelo método Varimax apresentou valores de 0,713 a 0,926, sendo apenas o item 2 alocado no terceiro componente.

**CONCLUSÕES:**

A versão brasileira é confiável e válida para uso no diagnóstico de ansiedade relacionada à realização de procedimentos ultrassonográficos no pré-natal. Devido à falta de correlação com o restante do construto, sugere-se a retirada do item 2 da versão final.

## INTRODUCTION

Anxiety is a debilitating emotional state or condition with the potential to alter the quality of life^1^. In addition to being the most prevalent psychiatric disorder, it is associated with several conditions that compromise the development of pregnancy and the fetus, from negative neonatal consequences (such as prematurity, low birth weight, low Apgar scores, fetal development deficit) to lasting effects on the physical and psychological development of children, as well as obstetric complications such as vaginal bleeding and the threat of abortion^2^. About one-fifth of the world's adult population has already suffered from some anxiety disorder at some point in their lives², with women being about 1.5 times more likely to develop them than men^3^. Brazil is the country with the highest rate of people with this type of disorder in the world. According to estimates by the World Health Organization, 9.3% of Brazilians have some anxiety disorder^4^. During the period before delivery, 9 to 22% of women have any of them^4^ ^,^ ^5^.

Maternal emotional state during prenatal care is associated with numerous adverse effects. The mental health of the pregnant woman is a risk factor for anxiety and postnatal depression^6^ ^,^ ^7^. For the child, maternal disorder increases the risk of anxiety, depression, attention deficit, hyperactivity and cognitive deficits in extrauterine life^8^ ^-^ ^15^. It may also result in infants too small for gestational age^16^ due to the excess of cortisol released by the pregnant woman^17^, abnormalities in several fetal tissues, and prematurity, aside from probably being related to low response of the fetal adaptive immune system to vaccines, which would explain the increase in infectious and autoimmune disease rates^17^ ^-^ ^19^. In addition, anxiety symptoms during pregnancy are related to the behavior of children in the first months postpartum: children of anxious mothers are more likely to show negative emotions^20^. Women with high levels of prenatal anxiety in early pregnancy are more likely to choose not to breastfeed and incur into early cessation of exclusive breastfeeding^21^ ^,^ ^22^.

The association between anxiety and prenatal diagnostic procedures is common, seeking to provide information on fetal gestation and development^23^. Obstetric ultrasonography, although not invasive, increases anxiety in pregnant women because of the potential to indicate fetal changes^24^. In Brazil, Febrasgo (Federação Brasileira das Sociedades de Ginecologia e Obstetrícia) instituted the use of at least three ultrasonography exams during pregnancy: first trimester morphological ultrasound, between 11 and 14 weeks; second trimester morphological ultrasonography and cervical uterine examination between 20 and 24 weeks; and obstetric ultrasonography between 34 and 36 gestation weeks^25^.

Several instruments are used to measure anxiety in women in the perinatal period, but only the Pregnancy Anxiety Scale^26^ was specifically designed for this use^27^. Nevertheless, none was developed to study anxiety prior to the conduction of diagnostic procedures, when concerns about fetal health and development are evident^23^.

Originated from a rigorous development process and from psychometric assessments, the Prenatal Diagnostic Procedures Anxiety Scale (PDPAS) is a tool that measures the patient's fear and anxiety before undertaking a prenatal diagnosis procedure, based on self-reported answers about her emotions and thoughts about the procedure itself and its results^23^. The PDPAS was recently created and translated into several languages, but validated only for the Croatian population; it is a sensitive method that can be used in research and screening in clinical settings^23^.

The original version of the PDPAS was developed by Košec et al.^23^, from the gynecology and obstetrics department of the University Hospital Center Sisters of Mercy in Zagreb, Croatia. The PDPAS was elaborated based on the clinical experience of the authors in gynecology, obstetrics and psychology, and was designed to be used before some procedures such as amniocentesis and ultrasonography^23^.

Currently, there is no validated instrument in Brazil that allows the evaluation of anxiety in these situations. This study aims to propose a Brazilian version and to observe its psychometric properties of reliability and construct validity. After adapting PDPAS to this cultural context, the Brazilian Portuguese version can be validated and used to measure the anxiety related to prenatal diagnostic procedures. It will also allow to widen the knowledge of health professionals and stimulate screening programs to avoid various complications for mothers and children. Therefore, the objective of this research is to cross-culturally adapt the PDPAS questionnaire for application in the Brazilian cultural context and to observe the psychometric properties of the proposed Brazilian version.

## METHODS

The consent of the authors of the original instrument was obtained in order to conduct this study, as well as for all stages of the translation process. The study was performed at the gynecology and obstetrics service of the Hospital Regional de São José (HRSJ), located in the city of São José, state of Santa Catarina, in the year of 2017.

The study was composed of two stages: one to propose the Brazilian version of PDPAS, and the second to observe its psychometric properties of reliability and validity.

### Step I - Proposal of the Brazilian Version of PDPAS

The guidelines for the process of cross-cultural adaptation are those recommended by Beaton et al.^28^ The direct translation of the PDPAS questionnaire from Croatian into Portuguese was carried out by two sworn translators, without medical knowledge, academic ties or knowledge of the purpose of the study. Both translations were compared and synthesized into a single version by the research authors. Based on this synthesis, a back-translation was performed by a native Croatian speaker, without any medical knowledge or of the original questionnaire.

A committee of experts evaluated the semantic, idiomatic, experimental and conceptual equivalence of the Brazilian version at this stage. The committee was constituted by two Ph.D. professors with experience in epidemiology and nursing, a doctorate student in health sciences, and a medical student. After identifying and discussing discrepancies in the process of translation and back-translation, a pre-final version was proposed. The pre-final version was applied in six pregnant women before an ultrasonography, in order to identify difficulties regarding the understanding of the questions or the layout and the time required for response. After analyzing the results of the pre-final version, the final version was approved (Annex).

### Step II - Evaluation of Psychometric Properties from the Brazilian Version of the PDPAS

A cross-sectional study was performed involving pregnant women awaiting obstetric ultrasonography scheduled at the gynecology and obstetrics service of the HRSJ, from March to June 2017. To calculate the sample size, a ratio of five pregnant women was used for each question, as recommended in the literature^29^. The total of 11 questions resulted in a sample of 55 patients selected by simple sampling. Patients aged 18 years or more and who knew how to read and write in the Portuguese language were included. Patients with clinical and psychiatric disorders that prevented participation in data collection and patients with an already known obstetric pathology were excluded. Data collection occurred by applying the Brazilian version of the PDPAS before the examination. At the same time, demographic and obstetric characteristics of the patients under study were collected and the Edinburgh Postnatal Depression Scale (EPDS)^30^ questionnaire translated and validated, in order to assess external reliability.

In the PDPAS, the interviewees are asked to score each item from 0 to 3, with 0 being “never or rarely” having thought about a certain subject, and 3 being “almost always or always” having thoughts about it. The scores are summed to achieve the final result, ranging from zero to 33. The EDPS, in turn, is a 10-question questionnaire designed to identify women with postpartum depression. The items from the scale correspond to various symptoms of clinical depression such as: guilt, sleep disorder, low energy, anhedonia, and suicidal ideas. The overall rating is made by the total score, which is the sum of the points of the 10 items. Higher scores indicate more depressive symptoms. The EPDS can be used within eight weeks after delivery and can also be used to track depression during pregnancy.

External reliability, meaning the correlation between two different instruments that evaluate the same domain, was calculated by Pearson's linear correlation and the intraclass correlation coefficient.

The psychometric property of internal consistency, a way to measure the correlation between different items of the same questionnaire, was obtained by Cronbach's alpha coefficient.

The psychometric property of validity was measured by means of exploratory factor analysis (EFA) with main component extraction (MCE). In order to observe the adequacy of EFA in the data of this study, the following previous analyses were made: relation between the number of questions in the questionnaire and the number of subjects interviewed, analysis of the correlation matrix between each pair of questions by Pearson's linear correlation, aside from the statistical tests Kaiser-Meyer-Olkin (KMO) and Bartlett's sphericity^29^.

For the definition of the number of factors, the criterion of latent dimensions, namely the Kaiser-Guttman criterion, was used, as it takes into account only the factors corresponding to eigenvalues higher than one (λ ≥ 1) or very close to one. For such, the MCE method was used, which minimizes the correlation between factors, the first one being formed by the highest percentage of the variance shared by the questions of the questionnaire. The use of this method allowed to perform the structural reduction of the data, to define rankings of observations by using factors, and to verify the validity of the previously established constructs.

Factor loadings were represented by Pearson correlation between the questions and each one of the factors. Based on the Kaiser-Guttman criterion, the factor loadings between the factors corresponding to eigenvalues smaller than one as well as corresponding to the questions must be low. Thus, the questions that shared small variance percentages with the others had their factor loadings elevated by a single factor. Conceptually, the sum of the squares of the loadings for each question should always be equal to the unit if the number of factors extracted equals the number of questions. This kind of sum is called commonality, which represents the shared total variance of each question in all factors based on the pre-selected eigenvalues. Commonality analysis allowed to verify whether any question did not share a significant percentage of variance with the factors defined.

The Varimax rotation method was used to minimize the number of questions that presented high loads in a given factor through the redistribution of loads and maximization of the variance shared in factors corresponding to smaller eigenvalues.

All analyzes were performed by means of the application SPSS, version 18.0. The research project was submitted to and approved by the Research Ethics Council of UNISUL, under Certificate of Presentation for Ethical Appreciation 62242916.9.0000.5369.

## RESULTS

The process of cross-cultural adaptation of the Croatian PDPAS instrument generated the version for the Brazilian Portuguese language (Annex).

For the cross-sectional study to observe its psychometric properties, 55 pregnant women were invited to participate. There was no withdrawal after the application of the questionnaires. The authors assumed the normal distribution of data, since in the psychometric evaluation process the analyses are performed between the same individuals.

The age of the pregnant women ranged from 18 to 41 years. The majority (70.9%) was between 20 and 35 years of age, Caucasian (72.7%), lived with partner (90.9%) and reported eight or more education years (67.3%). The gestational age of the participants at the time the questionnaire was applied ranged from 8 to 38 complete weeks of gestation, with average of 27±9 completed weeks. The obstetric characteristics of the patients evaluated are shown in Table 1.

**Table 1:** Obstetric characteristics of the study population. Hospital Regional de São José, state of Santa Catarina, Brazil, 2017. (n = 55)

Obstetric characteristics		n	%
Previous vaginal births			
	Yes	27	49.1
	No	28	50.9
Previous Cesarean section			
	Yes	16	29.1
	No	39	70.9
Previous abortions			
	Yes	13	23.6
	No	42	76.4
Previous gestational disease			
	Yes	13	23.6
	No	42	76.4
Previous fetal malformation			
	Yes	4	7.3
	No	51	92.7
Previous neonatal death			
	Yes	5	9.1
	No	50	90.9
Having performed ultrasonography in this gestation			
	Yes	51	92.7
	No	4	7.3

At the moment of evaluation, the patients waited for transvaginal (9.1%), morphological (34.5%), morphological with transvaginal (7.3%) or obstetric (49.1%) ultrasound examinations.

Data analysis showed a variance of the PDPAS score from zero to 29, reaching an average of 13.3±7.8 points. The EPDS^30^ ranged from zero to 20, with an average of 8.8±5.0 points, while its anxiety subscale presented scores ranging from zero to 12, with an average of 5.5±3.1 points.

The external reliability of the PDPAS was assessed by comparing it with the anxiety subscale from EPDS^30^, obtaining a Pearson correlation index of 0.404 (p = 0.002) and the intraclass correlation coefficient of R = 0.575 (95%CI 0.272–0.752).

The PDPAS internal consistency analysis determined a Cronbach's alpha of 0.886. Cronbach's alpha values, in case each of the questions was excluded, were similar to the total value for the 10 questions, except for question 2 (0.183) (Table 2).

**Table 2:** Reliability analysis by Cronbach's alpha of the Brazilian version of the Prenatal Diagnostic Procedures Anxiety Scale (PDPAS). Hospital Regional de São José, state of Santa Catarina, Brazil, 2017. (n = 55)

Items from the Brazilian version of PDPAS		Correlation of total item if corrected	Cronbach Alpha if item is deleted
1	I am afraid that the procedure will harm the baby.	0.526	0.888
2	Baby certainly has anomalies.	0.183	0.903
3	I am worried about the procedure itself.	0.677	0.879
4	I am worried about the result of the procedure.	0.718	0.876
5	I am afraid of pain during the procedure.	0.473	0.891
6	I am afraid of discomfort during the procedure.	0.594	0.884
7	The waiting for the results makes me anxious.	0.764	0.873
8	The waiting for the procedure is upsetting itself.	0.782	0.871
9	I am afraid of miscarriage due to the procedure.	0.568	0.886
10	I am afraid there is something wrong with the baby.	0.694	0.878
11	I am anxious because I do not know what to do if the results are abnormal.	0.735	0.875

The previous analysis of data suitability for EFA showed that the correlation matrix between the items presented significant results among the majority of crosses (p < 0.001), whereas Pearson correlation showed coefficients above 0.3. The KMO criterion for sample adequacy was 0.810, indicating a correlation between the variables. The Bartlett sphericity test also demonstrated suitability of the data for the EFA technique (p < 0.001). These measures showed that EFA could be performed. The communities showed that the percentage of explained variance of all items was higher than 50.0%.

Kaiser-Guttman's latent dimension criterion allowed for a reduction in the correlation between factors, defining three of them as responsible for explaining 78.5% of the total variance of the instrument for eigenvalues greater than or close to one. The eigenvalue results for the three major components are presented in Table 3. Likewise, the generated Scree plot demonstrated the factor groupings, with reduction in three main components at the point of inflection, according to the Figure.

**Figure:**
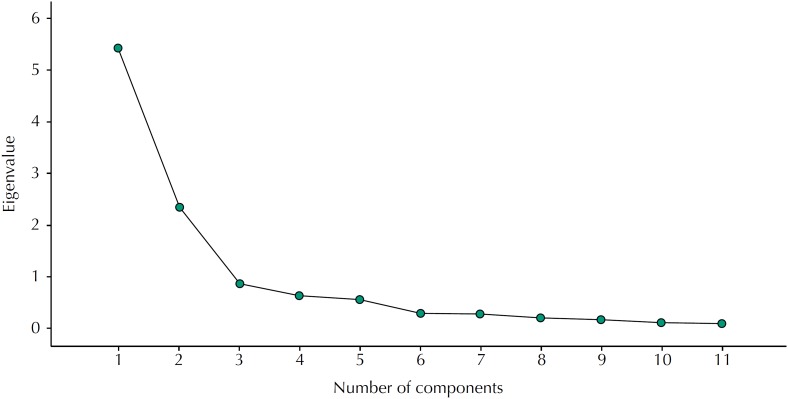
Scree plot. Hospital Regional de São José, state of Santa Catarina, Brasil, 2017. (n = 55)

**Table 3:** Total variance explained by the extraction method of principal component analysis from the Brazilian version of Prenatal Diagnostic Procedures Anxiety Scale. Hospital Regional de São José, state of Santa Catarina, Brazil, 2017. (n = 55)

Component	Extraction sum of squared loadings			Rotation sum of squared loadings		
	Total	% variance	% cumulative	Total	% variance	% cumulative
Component 1	5.410	49.186	49.186	4.098	37.251	37.251
Component 2	2.352	21.385	70.571	3.210	29.183	66.434
Component 3	0.869	7.896	78.468	1.324	12.033	78.468

The extraction of the main components with the Varimax rotation method presented values ranging from 0.713 to 0.926, and only item 2 was allocated to the third component, as shown in Table 4. The overall adjustment of the EFA was performed without item 2, demonstrating a strengthening of the weights charged to the remaining items.

**Table 4:** Analysis of the factorial components of each item obtained by the Varimax Rotation Method. Hospital Regional de São José, state of Santa Catarina, Brasil, 2017. (n = 55)

PDPAS Items		Extraction of principal components		
		1	2	3
10	I am afraid there is something wrong with the baby.	0.888		
11	I am anxious because I do not know what to do if the results are abnormal.	0.868		
7	The waiting for the results makes me anxious.	0.847		
4	I am worried about the result of the procedure.	0.776		
8	The waiting for the procedure is upsetting itself.	0.713		
3	I am worried about the procedure itself.	0.646		
6	I am afraid of discomfort during the procedure.		0.926	
5	I am afraid of pain during the procedure.		0.915	
9	I am afraid of miscarriage due to the procedure.		0.754	
1	I am afraid that the procedure will harm the baby.		0.693	
2	Baby certainly has anomalies.			0.842

PDPAS: Prenatal Diagnostic Procedures Anxiety Scale

## DISCUSSION

The cross-cultural adaptation process generated a Brazilian version of the PDPAS that was easy to apply before the ultrasound examinations in pregnant women. The application of the instrument to patients who performed amniocentesis was not possible due to the absence of this test in the routine of the service where this study was done, as it is uncommon in Brazil. In developed countries, especially where interruption of pregnancy is covered by local laws and culture, examination has much broader indications than those found in our setting.

External reliability was observed by the correlation between the Brazilian version of the PDPAS questionnaire and a subscale of the EPDS^30^ that presents a domain related to the evaluation of anxiety. The values found in the Pearson correlation analysis showed a weak correlation with the already validated instrument, but the intraclass correlation coefficient showed a satisfactory correlation between the two instruments. EPDS^30^ was originally developed to assess postnatal depression, but some of its items address anxiety, which allows this instrument to also be used for this domain.

The internal consistency, expressed by the Cronbach's alpha of 0.886, reached a high level, higher than the value found in the validation of the original instrument in Croatian (0.800)^23^. Then, each item of the instrument was evaluated, resulting in a variance from 0.183 to 0.782. Only item 2 presented low consistency on the set of questions, being outside the ideal standard. The removal of this question from the instrument changed the Cronbach's alpha value to 0.903, which demonstrates low reliability of this item in the general context of the construct.

In the validity analysis from the Varimax rotation method, it was possible to observe which items were grouped in each of the components. The first one consisted of questions 3, 4, 7, 8, 10 and 11; the second consisted of questions 1, 5, 6 and 9; the third consisted only of item 2. This grouping of items allowed to assess the domains that each component evaluated. Thus, the first component was related to anxiety and concern about the ultrasound procedure or an altered outcome of the examination. The second component was related to the pregnant woman's fear about questions related to the procedure itself. The third, containing only item 2, “The baby certainly has anomalies (malformation)”, which brings an already defined affirmation to the patient, was not related to the set of remaining items.

The original Croatian PDPAS^23^ showed the existence of only two dimensions, one related to fear of the procedure itself, comprising items 1, 3, 5, 6, 7, 8 and 9, and another to fear of abnormal results and concerns about fetal health, covering items 2, 4, 10 and 11. It is observed that the items constituting the dimensions of the Croatian instrument differ from those presented in the Brazilian version, even when compared to the first two components found in the present study.

As for the item 2 of the questionnaire, its permanence in the Brazilian version of the PDPAS should be questioned, since it was the only one to express consistency below 0.400, not reaching the minimum value to be considered reliable. In addition, item 2 was responsible for creating a third component in the questionnaire, presenting a different idea from the remaining items. Because it is a question that brings to mind a very limited thought that is not related to the anxiety of a diagnosis that is still to come, but rather to something already known, it is coherent and timely to propose its withdrawal from the PDPAS instrument in its Brazilian version.

Unlike the original study^23^, which limited patients to those who underwent ultrasonography in the second trimester of gestation, this Brazilian version covered all gestational ages and types of ultrasonography that can be performed during pregnancy. This difference was important to allow the Brazilian instrument to be used in any circumstance where there may be anxiety, without the need for alterations.

One limitation of the present study was the difficulty found to test the Brazilian version of the PDPAS in two different moments. Since the questionnaire was used with patients from the public healthcare network, it was not possible to perform a second application. Thus, it was not possible to perform test-retest reliability analysis, which would lead to additional knowledge for the research.

This study concluded that the Brazilian version of the PDPAS is reliable and valid, which allows its use to consolidate the diagnosis of anxiety during pregnancy, linked to the performance of ultrasonographic procedures in the prenatal period, which can either be detrimental to the mother as for its concept. There are still no published studies that use the PDPAS instrument to assess prenatal anxiety as well as analyze its importance in this diagnosis and role in reducing maternal-fetal risks and improving the quality of maternal life, but we believe that this can happen when its use is popularized. It is also suggested that question 2 be withdrawn from the final version of the instrument, given its lack of correlation with the rest of the instrument. Nevertheless, there is a need for further studies with other populations and situations in order to observe the behavior of the instrument and consolidate its use in Brazil.

## Annex Final version of the Prenatal Diagnostic Procedures Anxiety Scale questionnaire in Brazilian Portuguese.

**Table:**

While waiting for the prenatal diagnostic procedure*, pregnant women have different feelings and thoughts. Here are listed some of them. Please read carefully each question and circle the number which best represents how often the particular thought occurs to you.					
		Never or rarely	Sometimes	Often	Mostly or always
1.	I am afraid that the procedure will harm the baby.	0	1	2	3
2.	Baby certainly has anomalies.	0	1	2	3
3.	I am worried about the procedure itself.	0	1	2	3
4.	I am worried about the result of the procedure.	0	1	2	3
5.	I am afraid of pain during the procedure.	0	1	2	3
6.	I am afraid of discomfort during the procedure.	0	1	2	3
7.	The waiting for the results makes me anxious.	0	1	2	3
8.	The waiting for the procedure is upsetting itself.	0	1	2	3
9.	I am afraid of miscarriage due to the procedure.	0	1	2	3
10.	I am afraid there is something wrong with the baby.	0	1	2	3
11.	I am anxious because I do not know what to do if the results are abnormal.	0	1	2	3

* If the questionnaire is administered only for the specific prenatal diagnosis procedure, the word “procedure”/”postupak” (cro.) can be replaced by the name of the procedure (i.e., amniocentesis, ultrasound…).
